# Return to Sport After Foot and Ankle Surgery Is Inconsistently Defined and Assessed: A Systematic Review

**DOI:** 10.1002/ars2.70100

**Published:** 2026-10-29

**Authors:** Konstantinos Oikonomou, Kush Savsani, Phillip Wyatt, John Cyrus, Brady Ernst, Albert T. Anastasio, Conor N. O’Neill, J. Brett Goodloe, Annunziato Amendola

**Affiliations:** ^1^ Virginia Commonwealth University School of Medicine Richmond Virginia U.S.A.; ^2^ Department of Orthopaedics University of Alabama at Birmingham Birmingham Alabama U.S.A.; ^3^ Department of Orthopaedics Virginia Commonwealth University Health System Richmond Virginia U.S.A.; ^4^ Department of Orthopaedics Duke University Health System Durham North Carolina U.S.A.

## Abstract

**Purpose:**

To conduct a systematic review of return‐to‐sport (RTS) definitions in the orthopaedic literature, addressing common operative athletic foot and ankle pathologies including ankle instability, Achilles tendon ruptures or tendinopathy, and fifth metatarsal fractures.

**Methods:**

A systematic review was performed in adherence to the Preferred Reporting Items for Systematic Reviews and Meta‐Analyses guidelines. Cochrane, Embase, Medline, and CINAHL databases were searched for studies published through March 2024. Inclusion criteria were studies in which subjects were involved in some level of sports and received surgery for 1 of 3 common sports‐related injuries: ankle instability, Achilles tendinopathy, and fifth metatarsal fracture. The primary outcome was each study's definition of RTS.

**Results:**

There were 145 articles that met all inclusion criteria. Of these, 73 articles investigated ankle instability, 56 investigated Achilles tendinopathy, and 16 investigated fifth metatarsal fracture. Seventy‐nine articles (54.5%) had an unclear definition of RTS with 66 articles (45.5%) reporting a precise definition. Sixty‐four articles (44.1%) provided an isolated binary metric reporting whether a patient returned to sport or failed to provide a method for determining the timepoint for RTS. Thirty‐eight (26.2%) of the articles used an objective start criterion (e.g., resumption of competition), followed by custom (n = 27, 18.6%) and standardized patient‐reported questionnaire use (n = 21, 14.5%).

**Conclusions:**

Most articles included in this systematic review did not provide an explicit definition of RTS or a clearly defined assessment methodology. When specified, RTS was assessed using objective events, custom questionnaires, or standardized patient‐reported outcome measures, highlighting substantial heterogeneity in both RTS definitions and assessment approaches across foot and ankle literature.

**Level of Evidence:**

Level IV, systematic review of Level I to IV studies.

## Graphical abstract

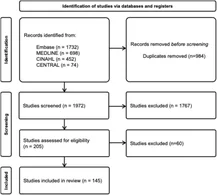

Injuries in sports are often framed in binary terms—either as minor or major.^1^, ^2^ Although minor injuries may require rest, rehabilitation, and conservative management, major injuries often require surgery and are typically described as season‐ending or career‐threatening. Injuries, especially at high levels of competition, are under significant scrutiny by the public, and returning players to their preinjury level is often seen as the only positive outcome. Likewise, it serves as a widely used benchmark in orthopaedic literature to assess recovery and successful intervention after injury or surgical procedures.^3^, ^4^

Despite its frequent use, return to sport (RTS) is not a standardized metric.^5^, ^6^, ^7^ For example, Hansen et al.^8^ studied the relation between the Achilles tendon Total Rupture Score and ability to return to work and sports and reported RTS as a primary outcome without clearly defining whether this constituted return to preinjury level or an alternate criterion. Moreover, Abdelatif and Batista^9^, ^10^ published 2 studies analyzing RTS in athletes after Achilles tendon rupture, although used different methods to determine RTS. One assessed RTS as resumption of competitive soccer games, whereas the other used a standardized patient‐reported outcome measure (PROM). If such variability exists within studies by the same authors, the inconsistency across the broader literature is potentially greater.

Foot and ankle pathologies constitute a substantial proportion of sports‐related injuries.^11^ This study focused on ankle instability, Achilles tendon pathology, and fifth metatarsal fractures because of their high prevalence as operative athletic foot and ankle pathologies. Previous systematic reviews have identified ankle injury as the most common foot and ankle injury in sports,^12^, ^13^ particularly those involving the anterior talofibular ligament. Likewise, Achilles tendon and metatarsal stress fracture injuries are the most common among tendon and stress fracture injuries.^13^ Understanding the prevalence of these injuries is crucial for developing targeted prevention strategies, optimizing surgical outcomes, and setting realistic RTS expectations for athletes across all levels.

Although RTS as a metric in concept is clearly a valuable consideration, inconsistencies in its definition limit the comparability of interventions across studies. This lack of standardization complicates clinical decision‐making and hinders the ability of athletes to set recovery expectations, increasing the risk of reinjury associated with premature returns. Clear, consistent benchmarks could help guide surgeons with decisions to initiate weight‐bearing, sport‐specific drills, and return to full competition, ultimately leading to safer and more individualized rehabilitation protocols. The purpose of this study is to conduct a systematic review of RTS definitions in the orthopaedic literature, addressing common operative athletic foot and ankle pathologies including ankle instability, Achilles tendon ruptures or tendinopathy, and fifth metatarsal fractures. We hypothesized that there would be significant discrepancies between subjective and objective definitions of RTS, accompanied by similar variability within each classification.

## METHODS

### Literature Search

A comprehensive search of peer‐reviewed, published literature was conducted in Medline, Embase, Cochrane, and CINAHL from the inception of the database to March 2024, with assistance from a professional librarian. Anatomic location was isolated from the following phrases and their counterparts: “foot” (“foot joint,” “foot fracture,” “foot injury,” and “feet”), “ankle” (“ankle injury,” “ankle joint,” “ankle fracture,” and “syndesmosis”), “toe” (“toe injury,” “toe fracture”), and “Achilles” (“Achilles tendon”). The desired population was isolated with the following phrases: “return to sport” (“return to play,” “return to activity,” “return to participation,” and “return to sports”), “orthopaedic procedures” (“orthopedic surgery,” “orthopaedic procedure,” “orthopedic procedures,” “orthopaedic surgery,” “surgical,” “surgeries,” repair*, and arthroscop*). Similar phrases were linked using the Boolean command “OR,” followed by combination with the Boolean command “AND” between anatomic location and desired population. Exact search terms and strategy are provided in Table S1.

Identified studies were imported to Covidence (Covidence Systematic Review Software), and duplicate articles were removed. The study selection process followed the Preferred Reporting Items for Systematic Reviews and Meta‐Analyses guidelines, with the selection process summarized in Figure 1. The full details of the protocol were prospectively registered on PROSPERO (CRD42024513573). Ethical review and approval were waived for this study owing to the analysis of previously published data. This work has not been previously published.

**FIGURE 1:**
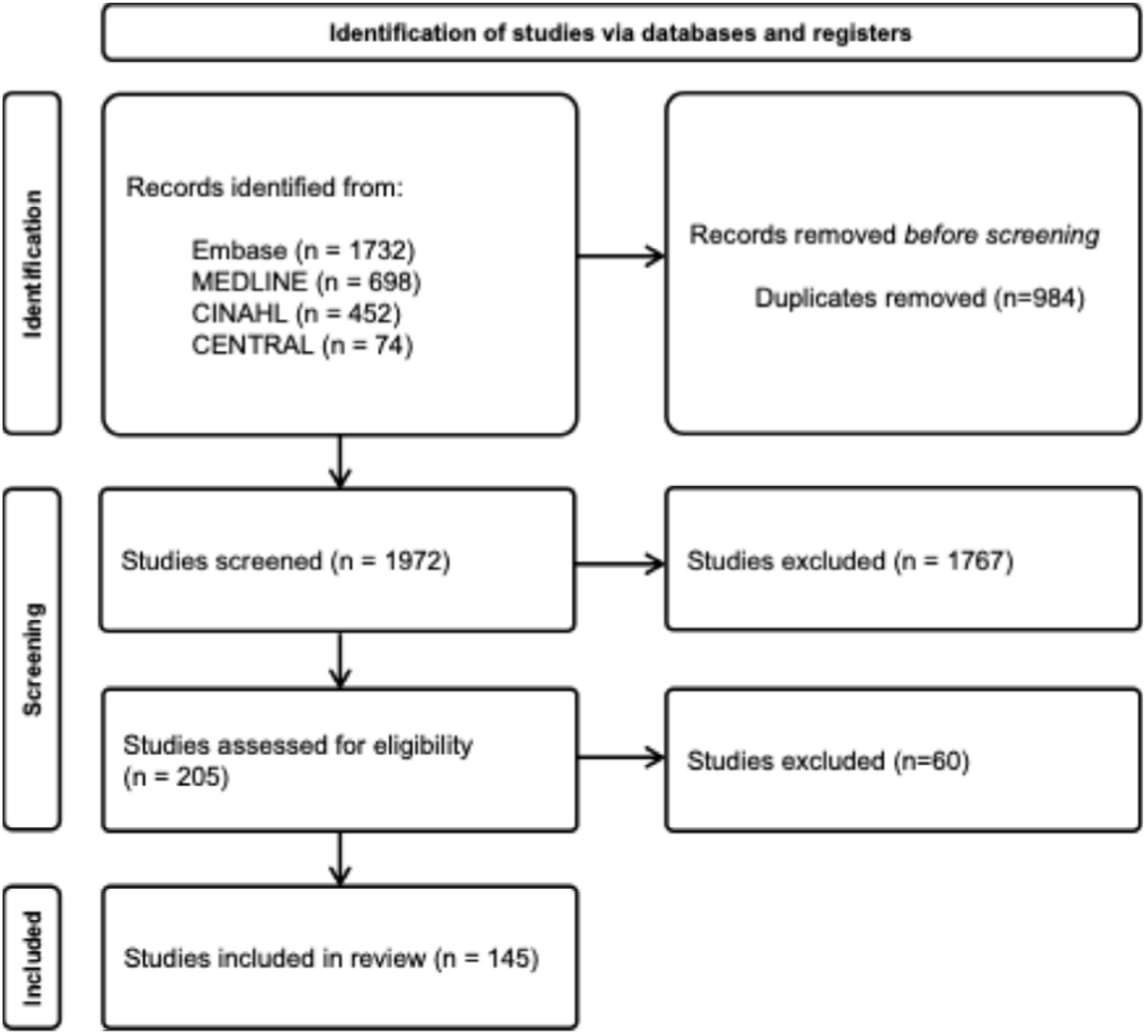
PRISMA 2020 flow diagram for systematic reviews. This flow diagram outlines included records identified from databases and registers, duplicates removed prior to screening, studies screened and excluded, and the final number of studies included in the review. (PRISMA, Preferred Reporting Items for Systematic Reviews and Meta‐Analyses.)

### Study Eligibility

Eligible studies were those that assessed postoperative RTS following surgical management of 1 of 3 common foot and ankle categories, including Achilles tendon pathology, ankle instability, and fifth metatarsal fractures. Achilles tendon pathologies included midportion or insertional Achilles tendinopathy and Achilles tendon ruptures. Ankle instability examined both acute (lateral, medial, combined, and syndesmotic) and chronic instability. Lastly, fifth metatarsal fractures included fractures involving either zones I, II, or III.

Studies were excluded if they focused on conditions outside these pathologies, used conservative treatment, or did not evaluate RTS as a primary outcome. The review included case series, retrospective cohort studies, and randomized controlled trials published prior to the date of database search. Non‐English articles, abstracts, systematic reviews, technical notes, letters to the editor, case reports, cross‐sectional studies, and surgical technique articles were excluded.

### Study Selection and Data Abstraction

Two independent reviewers (K.O., K.S.) screened all titles and abstracts for eligibility using the Covidence software. Full‐text review was subsequently completed using the same process. Discrepancies or conflicts at either stages were reviewed and resolved through discussion with the third reviewer (P.W.).

### Risk of Bias Assessment

A risk of bias assessment was not conducted for this study because the primary aim of this systematic review was to characterize RTS definitions and assessment methodologies, rather than to evaluate outcome validity or treatment efficacy.

### Data Extraction

Data from each included study was extracted independently by 2 reviewers into a Google Sheets spreadsheet (Google LLC, Mountain View, CA) with any discrepancies resolved by consensus or through a third reviewer if necessary. Extracted data included study design, level of evidence, pathology evaluated, and RTS assessment methodology. The primary outcome was the presence of an explicit or unclear definition of RTS. Secondary outcomes included the method used to determine RTS.

### Data Analysis

An RTS definition was considered precise or clear if a study clearly stated phrases such as “RTS was defined as …” or “RTS was determined by …”. Studies that did not provide such a statement, or listed RTS as a primary endpoint without describing the supporting methodology, were classified as unclear definitions.

RTS assessment methodologies were further categorized as objective events, custom questionnaires, standardized PROMs, or undefined. Objective events included predefined clinical or competitive milestones (e.g., first competitive game played after surgery or physician clearance). Custom questionnaires consisted of study‐specific patient‐reported assessments that directly queried RTS (e.g., “Have you returned to sport”). Standardized PROMs included validated instruments as surrogates for RTS (e.g., Tegner Activity Scale). Studies reporting only time‐based endpoints or binary RTS outcomes without methodology justification were classified as undefined.

Outcomes were summarized as counts and proportions. Primary and secondary outcomes were stratified by orthopaedic pathology category.

## RESULTS

### Literature Search and Screening

An initial search of Medline, Embase, Cochrane, and CINAHL databases returned 1972 studies after removing duplicates. Titles and abstracts were initially screened against the inclusion and exclusion criteria, after which 205 studies remained. The full texts of the remaining studies were then screened, where an additional 60 studies were excluded. Data extraction was performed on 145 studies. All results were reported in conformity with the Preferred Reporting Items for Systematic Reviews and Meta‐Analyses reporting standards. Most studies included were case series (n = 75 studies, 51.7% of all studies), followed by retrospective cohort studies (n = 49 studies, 33.8% of all studies). A summary of all included articles with associated level of evidence may be found in Table S2.^14^, ^15^, ^16^, ^17^, ^18^, ^19^, ^20^, ^21^, ^22^, ^23^, ^24^, ^25^, ^26^, ^27^, ^28^, ^29^, ^30^, ^31^, ^32^, ^33^, ^34^, ^35^, ^36^, ^37^, ^38^, ^39^, ^40^, ^41^, ^42^, ^43^, ^44^, ^45^, ^46^, ^47^, ^48^, ^49^, ^50^, ^51^, ^52^, ^53^, ^54^, ^55^, ^56^, ^57^, ^58^, ^59^, ^60^, ^61^, ^62^, ^63^, ^64^, ^65^, ^66^, ^67^, ^68^, ^69^, ^70^, ^71^, ^72^, ^73^, ^74^, ^75^, ^76^, ^77^, ^78^, ^79^, ^80^, ^81^, ^82^, ^83^, ^84^, ^85^, ^86^, ^87^, ^88^, ^89^, ^90^, ^91^, ^92^, ^93^, ^94^, ^95^, ^96^, ^97^, ^98^, ^99^, ^100^, ^101^, ^102^, ^103^, ^104^, ^105^, ^106^, ^107^, ^108^, ^109^, ^110^, ^111^, ^112^, ^113^, ^114^, ^115^, ^116^, ^117^, ^118^, ^119^, ^120^, ^121^, ^122^, ^123^, ^124^, ^125^, ^126^, ^127^, ^128^, ^129^, ^130^, ^131^, ^132^, ^133^, ^134^, ^135^, ^136^, ^137^, ^138^, ^139^, ^140^, ^141^, ^142^, ^143^, ^144^, ^145^, ^146^, ^147^

### Study Characteristics

Of the 145 studies, 16 (11.0%) were focused on fifth metatarsal fracture, 56 (38.6%) were focused on Achilles tendon pathology, and 73 (50.3%) were focused on ankle instability. Overall, 66 (45.5%) articles were deemed to have a clear definition of RTS, whereas 79 (54.5%) articles were deemed to have no clear definition. The complete distribution of definition clarity by article focus can be seen in Table 1.

**TABLE 1:** Study Classification and “Return‐to‐Sport” Definition Categorization

	**Definition Clear, n (%)**	**Unclear Definition, n (%)**	**Total, n (%)**
Fifth metatarsal fracture	11 (7.59)	5 (3.45)	16 (11.0)
Achilles tendon rupture	26 (17.9)	30 (20.7)	56 (38.6)
Ankle instability	29 (20.0)	44 (30.3)	73 (50.3)
Total	66 (45.5)	79 (54.5)	145

### Definitions of “RTS”

Table 2 shows the distribution of methods of defining RTS, with Figure 2 displaying the classification of each study within each injury type. Overall, 64 (44.1%) articles provided an isolated binary metric reporting whether a patient returned to sport or failed to provide a method for determining the timepoint for RTS. Time to RTS was determined by patient questionnaire during follow‐up in 27 (18.6%) of the articles. Huang et al. reported using both Foot and Ankle Ability Measure and Foot and Ankle Outcome Score (FAOS) scores in determining the timepoint for RTS.^148^ Boutelle et al. reported using both FAOS and Marx scores in determining timepoint for RTS.^149^

**TABLE 2:** Methods of Defining “Return to Sport”

	**Undefined, n (%)**	**Custom Questionnaire, n (%)**	**Objective Event, n (%)**	**Standardized PROM, n (%)**
Fifth metatarsal fracture	8 (5.52)	1 (0.690)	7 (4.83)	0
Achilles tendon	24 (16.6)	10 (6.90)	13 (8.97)	11 (7.59)
Ankle instability	32 (22.1)	16 (11.0)	18 (12.4)	10 (6.90)
Total	64 (44.1)	27 (18.6)	38 (26.2)	21 (14.3)

*Note*: Summated percentages may exceed 100% because of studies reporting multiple outcome measures. *Undefined*—binary metric (returning to sport or not)/time‐based endpoint with unclear definition. *Custom questionnaire*—PROM designed by authors of primary study. *Objective event*—defined endpoint marking return to sport, i.e., first game played, physician clearance, etc. *Standardized PROM*—standardized PROM, i.e., FAOS, TAS, FAAM, etc.

FAAM, Foot and Ankle Ability Measure; FAOS, Foot and Ankle Outcome Score; PROM, patient‐reported outcome measure; TAS, Tegner Activity Score.

**FIGURE 2:**
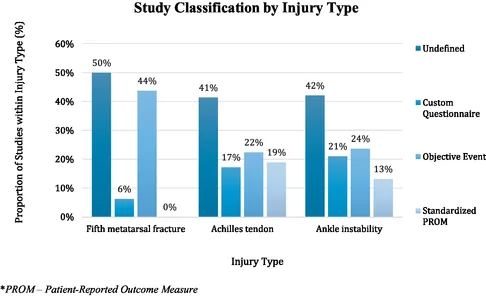
Graph of study classification by injury type. Bars represent the proportion (%) of studies within each injury type categorized by the method used to assess athletes’ return to sport. (PROM, patient‐reported outcome measure.)

Questionnaires were classified as the subjective interpretation of RTS by the patient. This included direct questioning on level of activity and participation in sports, as well as questioning about level of pain when playing sports. Eleven studies (7.6%) that reported RTS classification via questionnaire provided no clear guidelines for patient interview. These studies were classified as having no clear definition of RTS despite stating that patients were given a questionnaire during follow‐up because no criteria for determining the timepoint of RTS were provided.

Thirty‐eight (26.2%) articles reported the RTS for high school, college‐level, or professional athletes in which they determined the timepoint for RTS based on objective start date criteria. This includes the first preseason or regular season game played after surgery, specification of competition level, return to training or practice, or the first game played after surgery.

Nine studies (6.2%) used the Tegner Activity Score to determine the timepoint for RTS. Four studies (2.8%) used the FAOS. Four studies used the Foot and Ankle Ability Measure. Four studies used other objective metrics, including Victorian Institute of Sport Assessment‐Achilles score, Marx Activity Rating Scale, and Lauge‐Hansen classification of ankle injury. The distribution of standardized orthopaedic PROMs can be found in Table 3.

**TABLE 3:** Standardized Patient‐Reported Outcome Measures

	**FAAM, n**	**FAOS, n**	**TAS, n**	**VISA‐A, n**	**Marx, n**	**Lauge‐Hansen, n**
Fifth metatarsal fracture	‐	‐	‐	‐	‐	‐
Achilles tendon	‐	2	7	2	‐	‐
Ankle instability	4	2	2	‐	1	1
Total	4	4	9	2	1	1

FAAM, Foot and Ankle Ability Measure; FAOS, Foot and Ankle Outcome Score; Lauge‐Hansen, Lauge‐Hansen classification of ankle injury; Marx, Marx Activity Rating Scale; TAS, Tegner Activity Score; VISA‐A, Victorian Institute of Sport Assessment‐Achilles score.

## DISCUSSION

The most important finding of this study was that most included articles (54.5%) failed to provide an explicit definition of RTS, with “undefined” methodologies being the most common assessment of RTS (44.1%). Among the remaining studies, the most common approach used objective events (26.2%), custom questionnaires (18.6%), and standardized PROMs (14.5%). The variability in RTS definitions displayed in this study is significant given its widespread use in orthopaedic literature, its influence on athlete expectations, and its role in clinical decision‐making.

Clearly defining RTS enhances study clarity and allows for more effective comparison across studies. However, only a minority of studies provided explicit RTS definitions, and considerable variability existed among them. For example, Robbins et al. defined RTS as “having played in a minimum of 1 game following injury,” creating an isolated binary metric to define operative success.^150^ Khalil et al. included time‐based metrics, measuring RTS as “the first professional game played after Achilles tendon rupture,” tying RTS directly to competitive participation.^151^ Similarly, Hong and Calder defined RTS as both returning to “full preinjury level professional, national or international sports” and the time to RTS as reported by medical staff, although their study did not specify whether the metric was dependent on game participation.^152^

Studies lacking explicit RTS definitions showed even greater variability in their criteria. For instance, Choi et al. reported ability to RTS as a primary outcome; yet in the full‐text analysis, this was only reported as time to “return to prior activities of daily living” rather than sports‐specific activity.^153^ Ettinger et al. reported a median RTS time of 22.7 weeks but did not specify what outcome characterized RTS, instead relying on the FAOS as their primary measured outcome.^154^ Similarly, a study by Ibrahim reported the time it took for patients to return to “sporting activities” but did not define RTS independently or specify a clear endpoint.^155^

Among PROMs, isolated generalized activity questionnaires such as the Tegner Activity Scale were the most common, assessing overall activity level in comparison with preinjury function. Meanwhile, foot and ankle‐specific PROMs were also frequently reported, including the Foot and Ankle Ability Measure, FAOS, and Victorian Institute of Sport Assessment‐Achilles Score. Only 1 study (Boutelle et al.) incorporated both a generalized activity and foot and ankle‐specific PROM.^149^ Although PROMs provide valuable insights into RTS, a single PROM may not capture the full spectrum of recovery. A combination of a generalized activity questionnaire and a foot/ankle‐specific PROM may offer a more comprehensive assessment, ensuring a more accurate evaluation of an athlete's readiness to return to competition and daily activities. However, the use of multiple PROMs may contribute to patient fatigue from lengthy assessments and increased time burden on physicians, potentially leading to inefficient use of health care resources without a clear consensus on which combination provides the most clinically meaningful insights.

Previous studies have sought to establish uniform criteria to standardize the assessment of postoperative athletic recovery. In a consensus statement at the First World Congress in Sports Physical Therapy, Ardern et al. conceptualized postinjury recovery as a continuum, progressing from return to participation to RTS and ultimately return to performance. Return to participation reflects resumption of physical activity at any level, including rehabilitation or activity below the athlete's RTS goal, whereas RTS denotes a return to the athlete's defined sport without necessarily achieving prior performance standards. Return to performance represents restoration of sport‐specific performance at or above preinjury level, often encompassing physical capacity as well as competitive effectiveness.^156^

Despite the availability of such consensus frameworks, substantial heterogeneity in RTS definitions and reporting persists, as shown by the findings of the present review. To our knowledge, only 1 other systematic review, conducted by Doege et al., has attempted to synthesize definitions of RTS across the orthopaedic literature.^157^ Their study included only studies providing explicit RTS definitions, analyzed a broad range of operative athletic injuries, and categorized RTS based on objective measures, such as participation in a competitive game, returning the next season, or resumption training. Notably, among the 29 studies included, the authors similarly identified marked heterogeneity, with only a minority incorporating competition‐level modifiers and majority relying on binary outcomes that distinguished only whether an athlete was able to RTS.

Our study uniquely examined literature with unclear or inconsistent RTS definitions, focusing specifically on common foot and ankle pathologies rather than all operative athletic injuries. As a result, our review included 145 studies, compared with the 29 studies analyzed by Doege et al. Additionally, although their study provided detailed categorizations of objective RTS measures, we grouped these into a broader “objective event” category for a more streamlined analysis. This wider lens highlights the variability and need for standardization across the entire body of foot and ankle literature, rather than attempting to precisely subclassify existing objective criteria.

RTS is a complex metric that requires a more standardized and comprehensive approach in orthopaedic literature. Future research should focus on improving the clarity, accuracy, and applicability of RTS definitions and assessments for patient outcomes and education.

One major challenge in the current literature is the lack of consistent RTS definitions. Many studies use varying criteria, ranging from physician clearance to actual game participation. To improve comparability across studies, future research should establish clear, standardized definitions that differentiate between return to training, physician clearance, and return to competition. Time‐based metrics for these stages would provide greater insight into an athlete's recovery timeline and functional progress. Furthermore, consideration of sport‐specific demands is essential. Very few studies appropriately differentiate RTS by sport demands. For example, soccer, American football, and basketball involve substantial risk to the lateral ankle ligamentous complex. Additional time to achieve full clearance after surgery for ankle instability in athletes participating in these sports may be indicated relative to sports such as track or swimming. Optimally, each sport would be considered in isolation across the literature for the most accurate RTS criteria, but the current literature lacks sample size and study numbers to provide granularity in this regard.

Most RTS studies do not assess return to performance, limiting their ability to measure true recovery. For professional athletes, inclusion of performance‐based statistics and reinjury rates could provide more objective insight into recovery quality. In contrast, incorporating data from wearable technology data (i.e., smartwatches) could provide an additional measure of objective performance data for the recreational athlete.

As in the clinical situation, RTS is best assessed through a combination of objective events (i.e., return to competition) and subjective patient‐reported outcomes. Although a dual approach between generalized activity and joint‐specific PROMs can provide a comprehensive assessment, this must be balanced against risks of patient fatigue and concerns about clinical efficiency.

Given the variability in RTS definitions, there is a need for consensus guidelines, potentially via Delphi studies, to establish standardized definitions specifically tailored to foot and ankle surgery. Standardizing RTS definitions could improve clinical decision‐making by allowing surgeons to more precisely tailor postoperative rehabilitation timelines with consistent evidence‐based criteria that comprehensively capture an athlete's functional and physiological readiness to return. Ultimately, it would support shared decision‐making, strengthen trust between physicians and patients, and potentially lead to better long‐term functional and competitive outcomes.

### Limitations

This study is not without limitations. The term “RTS” may encompass a range of intended meanings, and the precise intent behind each author's definition may not be fully ascertainable from the text alone. This linguistic variability introduces some subjectivity into the categorization of RTS definitions; however, our use of predefined criteria and independent reviewer consensus was intended to enhance consistency and reproducibility. Additionally, this systematic review focused on a specific subset of foot and ankle pathologies, which may limit the generalizability of these findings to the broader foot and ankle literature and orthopaedics as a whole. Lastly, substantial heterogeneity among the included studies limited the feasibility of conducting a meta‐analysis.

## CONCLUSIONS

Most articles included in this systematic review did not provide an explicit definition of RTS or a clearly defined assessment methodology. When specified, RTS was assessed using objective events, custom questionnaires, or standardized PROMs, highlighting substantial heterogeneity in both RTS definitions and assessment approaches across foot and ankle literature.

## SUPPORTING INFORMATION

Additional supporting information can be found online in the Supporting Information section.

## DISCLOSURES

The authors (J.B.G., A.A.) declare the following financial interests/personal relationships which may be considered as potential competing interests: J.B.G. reports support provided by VCU Health System; reports a relationship with VCU Health System. A.A. reports a relationship with Arc Techtonics, Rubber City Bracing, Springer, and Wolter Kluwer that include funding grants; Bone Solutions and Miach Orthopaedics that include equity or stocks; Arthrex that includes nonfinancial support; and American Academy of Orthopaedic Surgeons and *American Journal of Sports Medicine* that include board membership. The other authors (K.O., K.S., P.W., J.C., B.E., A.T.A., C.N.O.) declare that they have no known competing financial interests or personal relationships that could have appeared to influence the work reported in this paper.

## Supporting information

Supplementary Material
