# Supplementary material for: Return to Sport After Foot and Ankle Surgery Is Inconsistently Defined and Assessed: A Systematic Review
Source: Arthrosc Sports Med Rehabil. 2026 Oct 29:e70100. Online ahead of print. doi: 10.1002/ars2.70100 (PMC13717671; doi:10.1002/ars2.70100)
Supplement: Supplementary file 1 [file ARS2-9999-e70100-s001.pdf]

SUPPORTING INFORMATION

Table S1. Literature Search Strategy Among Selected Databases

Embase (Ovid) Strategy

ID	Strategy	Hits
1	exp foot joint/ or exp foot fracture/ or exp foot/ or exp foot injury/	93691
2	exp ankle injury/ or exp ankle joint/ or exp ankle/ or exp ankle fracture/	60256
3	exp toe/ or exp toe injury/ or exp toe fracture/	18378
4	(foot or feet or toe or toes or ankle or ankles or syndesmosis or "achilles tendon").ti,ab,kf.	289702
5	1 or 2 or 3 or 4	325089
6	exp orthopedic surgery/	605093
7	("orthopedic surgery" or "orthopaedic procedure" or "orthopedic procedures" or "orthopaedic surgery" or surgery or surgeries or surgical or repair* or arthroscop*).ti,ab,kf.	3439546
8	6 or 7	3719499
9	exp return to sport/ or (return* adj3 (athlet* or sport* or play* or activit* or participat*)).ti,ab,kf.	26064
10	5 and 8 and 9	1732

Medline (Ovid) Strategy

(exp Foot/ or exp "Foot Joints"/ or exp "Foot Injuries"/ or exp "Ankle Joint"/ or exp Ankle/ or exp "Ankle Fractures"/ or exp "Ankle Injuries"/ or (foot or feet or toe or toes or ankle or ankles or syndesmosis or "achilles tendon").ti,ab,kf.) and (exp "Orthopedic Procedures"/ or ("orthopedic surgery" or "orthopaedic procedure" or "orthopedic procedures" or "orthopaedic surgery" or surgery or surgeries or surgical or repair* or arthroscop*).ti,ab,kf.) and (exp "return to sport"/ or ("return to sport" or "return to sports" or "return to play" or "return to activity" or "return to activities" or "return to participation").ti,ab,kf.)	698

Cochrane Strategy

(([mh Foot] OR [mh "Foot Joints"] OR [mh "Foot Injuries"] OR [mh "Ankle Joint"] OR [mh Ankle] OR [mh "Ankle Fractures"] OR [mh "Ankle Injuries"] OR foot OR feet OR toe OR toes OR ankle OR ankles OR syndesmosis OR "achilles tendon") AND ([mh "Orthopedic Procedures"] OR ("orthopedic surgery" OR "orthopaedic procedure" OR "orthopedic procedures" OR "orthopaedic surgery" OR surgery OR surgeries OR surgical OR repair* OR arthroscop*))) AND ([mh "return to sport"] OR "return to sport" OR "return to sports" OR "return to play" OR "return to activity" OR "return to activities" OR "return to participation")	88

*CINAHL Strategy *restricted to academic journals*

(((MH Foot+) OR (MH "Foot Joints+") OR (MH "Foot Injuries+") OR (MH "Ankle Joint+") OR (MH Ankle+) OR (MH "Ankle Fractures+") OR (MH "Ankle Injuries+") OR foot OR feet OR toe OR toes OR ankle OR ankles OR syndesmosis OR "achilles tendon") AND ((MH "Orthopedic Procedures+") OR ("orthopedic surgery" OR "orthopaedic procedure" OR "orthopedic procedures" OR "orthopaedic surgery" OR surgery OR surgeries OR surgical OR repair* OR arthroscop*))) AND ((MH "return to sport+") OR "return to sport" OR "return to sports" OR "return to play" OR "return to activity" OR "return to activities" OR "return to participation")	452

Table S2. Summary of Included Articles

**Author (Year) [Ref]**	**Pathology Examined**	**LOE**	**Definition Type**	**Definition Category**

Abdelatif & Batista (2021) [9]	Achilles Tendon	2	Precise	Objective Event
Abdelatif & Batista (2021) [10]	Achilles Tendon	3	Precise	Tegner
Ahn & Jeong (2022) [14]	Achilles Tendon	4	Precise	Tegner
Ahn et al. (2013) [15]	Ankle Instability	3	Unclear	Unclear Method
Ajaka et al. (2022) [16]	Ankle Instability	2	Unclear	Unclear Method
Alfredson et al. (2018) [17]	Achilles Tendon	4	Unclear	Custom Q and Objective Event and VISA-A
Baumbach et al. (2017) [18]	Fifth Metatarsal Fracture	2	Unclear	Unclear Method
Baumfield et al. (2021) [19]	Fifth Metatarsal Fracture	4	Precise	Unclear Method
Bell et al. (2013) [20]	Achilles Tendon	1	Precise	Custom Q
Bernstein et al. (2018) [21]	Fifth Metatarsal Fracture	4	Precise	Objective Event
Boutelle et al. (2023) [149]	Ankle Instability	4	Precise	FAOS and Marx Activity Score
Bouveaua et al. (2022) [22]	Ankle Instability	4	Precise	Unclear Method
Boykin et al. (2010) [23]	Ankle Instability	4	Unclear	Unclear Method
Calder et al. (2016) [24]	Ankle Instability	2	Unclear	Unclear Method
Campillo-Recio et. al. (2023) [25]	Achilles Tendon	2	Unclear	Unclear Method
Carpenter et al. (2020) [26]	Achilles Tendon	4	Unclear	Unclear Method
Cerulli et al. (1992) [27]	Ankle Instability	4	Unclear	Unclear Method
Chiu et al. (2013) [28]	Achilles Tendon	4	Unclear	Unclear Method
Choi et al. (2013) [153]	Fifth Metatarsal Fracture	4	Unclear	Unclear Method
Coetzee et al. (2018) [29]	Ankle Instability	4	Unclear	Objective Event and FAAM
Colvin et al. (2009) [30]	Ankle Instability	3	Precise	Custom Q
D'Hooghe et al. (2020) [31]	Ankle Instability	2	Precise	Objective Event
Deng et al. (2023) [32]	Achilles Tendon	1	Unclear	Unclear Method
Doğar et al. (2021) [33]	Ankle Instability	1	Unclear	Unclear Method
Du et al. (2023)[34]	Ankle Instability	3	Unclear	Custom Q
Ettinger et al. (2015)[154]	Achilles Tendon	4	Unclear	FAOS
Feng et al. (2021) [35]	Ankle Instability	3	Precise	Unclear Method
Feng et al. (2022) [36]	Ankle Instability	3	Unclear	Objective Event
Feng et al. (2020) [37]	Ankle Instability	3	Unclear	Unclear Method

Feng et al. (2022) [38]	Ankle Instability	3	Unclear	Unclear Method
Feng et al. (2022) [39]	Ankle Instability	3	Unclear	Unclear Method
Feng et al. (2023) [40]	Ankle Instability	3	Unclear	Unclear Method
Ferkel et al. (1991) [41]	Ankle Instability	3	Unclear	Objective Event
Ficek et al. (2021) [42]	Fifth Metatarsal Fracture	4	Precise	Objective Event
Gamal et al. (2021) [43]	Achilles Tendon	4	Unclear	Unclear Method
Ghasemi et al. (2023) [44]	Ankle Instability	4	Precise	Custom Q
Grassi et al. (2020) [45]	Achilles Tendon	4	Precise	Objective Event
Guillo et al. (2013) [46]	Achilles Tendon	2	Precise	Custom Q
Hanada et al. (2019) [47]	Ankle Instability	4	Unclear	Unclear Method
Hansen et al. (2015) [8]	Achilles Tendon	2	Unclear	Unclear Method
Hardy et al. (2018) [48]	Achilles Tendon	4	Precise	Custom Q
Harris et al. (2022) [49]	Ankle Instability	2	Unclear	Unclear Method
Hodgens et al. (2021) [50]	Achilles Tendon	3	Precise	Objective Event
Holzgrefe et al. (2020) [51]	Achilles Tendon	3	Precise	Tegner
Hong & Calder (2023) [152]	Ankle Instability	3	Precise	Objective Event
Hong et al. (2016) [52]	Achilles Tendon	4	Precise	Tegner
Hong et al. (2023) [53]	Ankle Instability	4	Unclear	Unclear Method
Hörterer et al. (2020) [54]	Achilles Tendon	4	Unclear	Custom Q
Hou et al. (2022) [55]	Ankle Instability	2	Unclear	Unclear Method
Hsu et al. (2015) [56]	Ankle Instability	4	Precise	Objective Event
Hsu et al. (2015) [57]	Achilles Tendon	3	Unclear	Unclear Method
Huang et al. (2023) [148]	Ankle Instability	4	Unclear	FAAM and FAOS
Hufner et. al. (2006) [58]	Achilles Tendon	3	Unclear	Custom Q
Ibrahim (2009) [155]	Achilles Tendon	4	Unclear	Unclear Method
Jack et al. (2017) [59]	Achilles Tendon	3	Precise	Unclear Method
Jain et al. (2022) [60]	Ankle Instability	4	Unclear	Unclear Method
Jallageas et al. (2013) [61]	Achilles Tendon	4	Precise	Unclear Method
Japjec et al. (2015) [62]	Fifth Metatarsal Fracture	3	Precise	Unclear Method
Jupiter et al. (2024) [63]	Ankle Instability	4	Precise	Objective Event
Kennedy et al. (2012) [64]	Ankle Instability	4	Unclear	Custom Q
Kerkhoffs et al. (2002) [65]	Achilles Tendon	4	Unclear	Unclear Method

Khalil et al. (2020) [151]	Achilles Tendon	3	Precise	Objective Event
Kim & Shin (2021) [66]	Ankle Instability	4	Precise	Objective Event
Kosaka et al (2011) [67]	Achilles Tendon	4	Unclear	Custom Q
Kramer et al. (2022) [68]	Achilles Tendon	4	Precise	Tegner
Kramer et al. (2010) [69]	Ankle Instability	4	Precise	Custom Q
Lahm et al. (1998) [70]	Ankle Instability	4	Unclear	Custom Q
Lerch et al. (2020) [71]	Achilles Tendon	3	Precise	Tegner
Letts et al. (2003) [72]	Ankle Instability	4	Unclear	Unclear Method
Li et al. (2019) [73]	Ankle Instability	3	Unclear	Objective Event
Li et al. (2020) [74]	Ankle Instability	3	Precise	Tegner
Li et al. (2022) [75]	Ankle Instability	3	Unclear	Unclear Method
Liu et al. (2022) [76]	Ankle Instability	3	Unclear	Unclear Method
Lopes et al. (2018) [77]	Ankle Instability	2	Unclear	Custom Q
Maffulli et al. (2006) [78]	Achilles Tendon	3	Precise	Custom Q
Maffulli et al. (2013) [79]	Ankle Instability	4	Precise	Custom Q
Maffulli et al. (2018) [80]	Achilles Tendon	4	Unclear	Unclear Method
Maffulli et al. (2021) [81]	Achilles Tendon	4	Unclear	Unclear Method
Maffulli et al. (2021) [82]	Achilles Tendon	2	Unclear	Unclear Method
Majewski et al. (2008) [83]	Achilles Tendon	2	Precise	Custom Q
Matsui et al. (2016) [84]	Ankle Instability	3	Precise	Objective Event
May et al. (2022) [85]	Ankle Instability	4	Precise	Custom Q
Metz et al. (2008)[86]	Achilles Tendon	1	Unclear	Unclear Method
Miller et al. (2018) [87]	Fifth Metatarsal Fracture	4	Precise	Objective Event
Miranda et al. (2020) [88]	Fifth Metatarsal Fracture	3	Unclear	Unclear Method
Miyamoto et al. (2014) [89]	Ankle Instability	3	Precise	Custom Q
Miyamoto et al. (2015) [90]	Ankle Instability	3	Unclear	Objective Event
Mologne et al. (2005) [91]	Fifth Metatarsal Fracture	1	Unclear	Unclear Method
Moradi & Cengiz (2021) [92]	Ankle Instability	4	Unclear	Unclear Method
Morimoto et al. (2021) [93]	Fifth Metatarsal Fracture	4	Precise	Custom Q
Murawski & Kennedy (2010) [94]	Ankle Instability	4	Unclear	Unclear Method
Murawski et al. (2011) [95]	Fifth Metatarsal	4	Precise	Objective Event

	Fracture			
Nakajima (2022) [96]	Achilles Tendon	4	Unclear	VISA-A
Nam et al. (2019) [97]	Achilles Tendon	3	Unclear	Unclear Method
Obut et al. (2017) [98]	Achilles Tendon	4	Unclear	FAOS
Ogilvie-Harris et al. (1997) [99]	Ankle Instability	4	Precise	Custom Q
Osbahr et al. (2013) [100]	Ankle Instability	4	Unclear	Unclear Method
Paez et al. (2021) [101]	Ankle Instability	3	Precise	FAAM
Pecina et al. (2011) [102]	Fifth Metatarsal Fracture	4	Unclear	Unclear Method
Peterson et al. (2019) [103]	Achilles Tendon	3	Precise	Tegner
Petrera et al. (2024) [104]	Ankle Instability	4	Unclear	Unclear Method
Porter et al. (2005) [105]	Fifth Metatarsal Fracture	4	Precise	Objective Event
Porter et al. (2005) [106]	Ankle Instability	4	Precise	Custom Q
Ready et al. (2021) [107]	Achilles Tendon	4	Precise	Objective Event
Robbins et al. (2022) [150]	Ankle Instability	3	Unclear	Objective Event
Robertson et al. (2014) [108]	Ankle Instability	3	Unclear	Lauge-Hansen Score
Rouvillain et al. (2010) [109]	Achilles Tendon	4	Unclear	Unclear Method
Rungprai & Phisitkul (2018) [110]	Achilles Tendon	4	Unclear	Unclear Method
Rungprai et al. (2016) [111]	Ankle Instability	3	Unclear	Unclear Method
Ryan & Rodriguez (2016) [112]	Ankle Instability	4	Precise	Custom Q
Sanchez et al. (2021) [113]	Achilles Tendon	3	Unclear	Unclear Method
Saxena (2003) [114]	Achilles Tendon	4	Precise	Objective Event
Saxena (2000) [115]	Ankle Instability	4	Unclear	Unclear Method
Stenquist et al. (2016) [116]	Ankle Instability	4	Precise	FAAM
Sigonney et al. (2021) [117]	Ankle Instability	2	Unclear	Unclear Method
Sochacki et al. (2019) [118]	Achilles Tendon	3	Precise	Objective Event
Steinmetz et al. (2016) [119]	Ankle Instability	4	Unclear	Unclear Method
Stone et al. (2021) [120]	Fifth Metatarsal Fracture	4	Precise	Objective Event
Su et al. (2023) [121]	Ankle Instability	1	Precise	Tegner
Su et al. (2023) [122]	Ankle Instability	3	Unclear	Unclear Method
Suchak et al. (2008) [123]	Achilles Tendon	1	Unclear	Unclear Method
Sugimoto et al. (2023) [124]	Ankle Instability	4	Precise	Objective Event

Sun et. al. (2020) [125]	Ankle Instability	4	Unclear	Custom Q
Takao et al. (2012) [126]	Ankle Instability	3	Precise	Objective Event
Takao et al. (2024) [127]	Ankle Instability	3	Unclear	Unclear Method
Talbot et al. (2012) [128]	Achilles Tendon	4	Unclear	Unclear Method
Teramoto et al. (2022) [129]	Ankle Instability	4	Unclear	Objective Event
Tramer et al. (2021) [130]	Achilles Tendon	3	Precise	Objective Event
Trofa et al. (2017) [131]	Achilles Tendon	3	Precise	Objective Event
Trofa et al. (2018) [132]	Achilles Tendon	3	Precise	Objective Event
Uchiyama et al. (2007) [133]	Achilles Tendon	4	Precise	Objective Event
Usuelli et al. (2017) [134]	Achilles Tendon	4	Unclear	Custom Q
Usuelli et al. (2020) [135]	Ankle Instability	4	Unclear	Custom Q
Watson et al. (2020) [136]	Fifth Metatarsal Fracture	4	Precise	Objective Event
Wever et al. (2022) [137]	Ankle Instability	4	Precise	Objective Event
White et al. (2016) [138]	Ankle Instability	3	Precise	Objective Event
Wise et al. (2020) [139]	Achilles Tendon	3	Unclear	Unclear Method
Won Lee et al. (2022) [140]	Achilles Tendon	4	Unclear	Unclear Method
Xiong et al. (2023) [141]	Ankle Instability	3	Precise	Unclear Method
Yang et al. (2019) [142]	Achilles Tendon	3	Precise	Objective Event
Yasuda et al. (2017) [143]	Ankle Instability	4	Precise	Objective Event
Yoo & Yang (2016) [144]	Ankle Instability	3	Unclear	Unclear Method
Young et al. (2020) [145]	Fifth Metatarsal Fracture	4	Precise	Unclear Method
Zaffagnini et al. (2018) [146]	Achilles Tendon	4	Unclear	Unclear Method
Zhou et al. (2021) [147]	Ankle Instability	3	Precise	Custom Q
